# Supplementary material for: A zirconium metal-organic framework with SOC topological net for catalytic peptide bond hydrolysis
Source: Nat Commun. 2022 Mar 11;13:1284. doi: 10.1038/s41467-022-28886-5 (PMC8917178; doi:10.1038/s41467-022-28886-5)
Supplement: Supplementary file 1 — Supplementary Information [file 41467_2022_28886_MOESM1_ESM.pdf]

Supplementary Information

A Zirconium Metal-Organic Framework with SOC Topological Net for Catalytic Peptide Bond Hydrolysis

Sujing Wang, Hong Giang T. Ly, Mohammad Wahiduzzaman, Charlotte Simms, Iurii Dovgaliuk, Antoine Tissot, Guillaume Maurin, Tatjana N. Parac-Vogt and Christian Serre

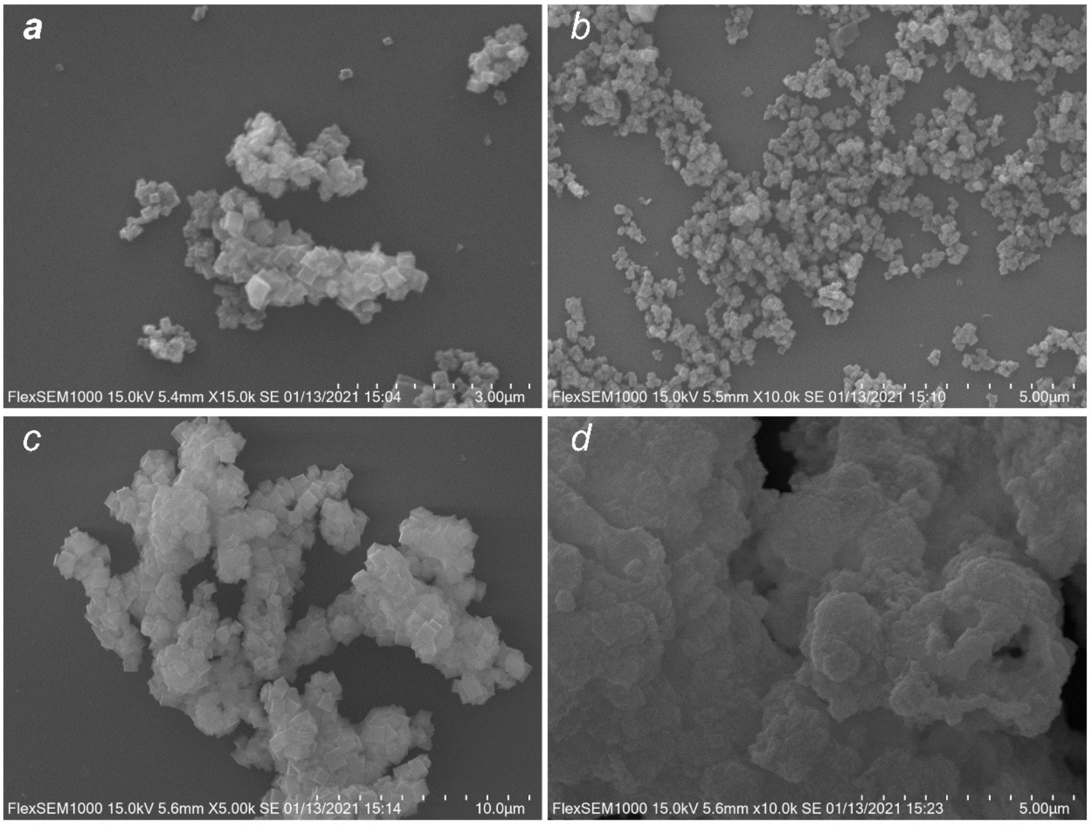

Supplementary Figure 1. SEM images of some representative MIP-201 products obtained under different reaction conditions. (a), Reaction condition of $ZrCl_4$ (115 mg, 0.5 mmol), H_4mdip (85 mg, 0.25 mmol) in acetic acid (5 mL) heated at 120 °C for 48 hours in autoclave. (b), Reaction condition of $ZrCl_4$ (460 mg, 2 mmol), H_4mdip (340 mg, 1 mmol) in acetic acid (10 mL) heated at 120 °C for 48 hours in autoclave. (c), Reaction condition of $ZrCl_4$ (230 mg, 1 mmol), H_4mdip (85 mg, 0.25 mmol) in formic acid (1 mL) and acetic anhydride (2 mL) heated at 120 °C for 48 hours in autoclave. (d), Reaction condition of $Zr(SO_4)_2{\cdot}4H_2O$ (10.8 g), H_4mdip (5.1 g) refluxed in water (300 mL) and acetic acid (100 mL) at 120 °C for 12 hours.

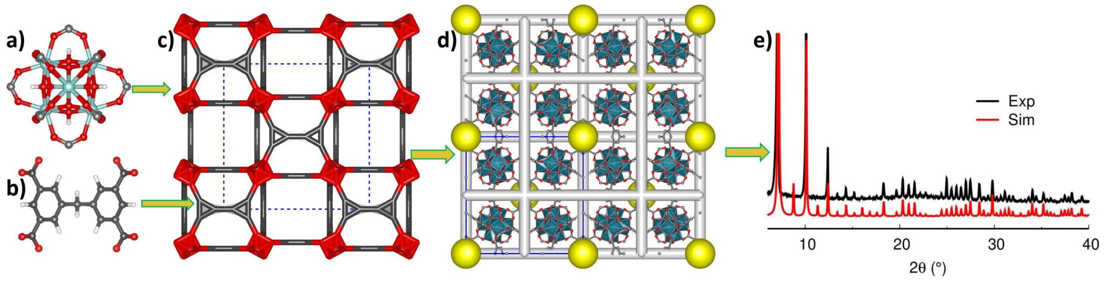

Supplementary Figure 2. Structure solution details. A Zr_6-oxo cluster inorganic building unit (a) and a tetracarboxylate mdip linker molecule (b) are used to decorate the vertices and edges of the cdj-a network topology (c) which results a primary model for MIP-201 framework (d). The corresponding simulated PXRD pattern calculated from the DFT optimized geometry compared with the experimental data presented in (e).

We adopted a topology guided reverse engineering approach[1, 2] to solve the crystal structure of MIP-201. In this procedure, at first, a rough structural model was derived from the application of direct methods to the experimental PXRD data. This led to the identification of the underlying topological network of the framework in which most of the metal centers unambiguously dictate a cdj-a soc type topology and associated to a cubic *I*m-3 (ITA number 204) system (*a*=24.7034 Å). A subsequent structural model was constructed by decorating the vertices and edges of the network by 6-connected Zr_6 SBUs and 4-connected mdip ligands, respectively (Supplementary Figure 2). The so-obtained structure was then geometry optimized at the Kohn-Sham density-functional theory (DFT) level using the Quickstep module[3] of the CP2K program[4, 5] which is based on Gaussian Plane Wave (GPW) formalism. We have employed the general gradient approximation (GGA) to the exchange-correlation functional according to Perdew-Burke-Ernzerhof (PBE)[6] in a combination of Grimme's DFT-D3 semi-empirical dispersion corrections.[7, 8] The interactions between core electrons and valence shells of the atoms were described by the pseudo-potentials derived by Goedecker, Teter, and Hutter (GTH-PPs).[9, 10, 11] We used the MOLOPT[12] Gaussian basis sets optimized for DFT calculations in molecular systems, which are also particularly designed for the use with GTH-PPs. Here, Triple-ζ plus valence polarized Gaussian-type basis sets (TZVP) were considered for all atoms, except for the Zr centers, where short ranged double-ζ plus valence polarization functions (DZVP) were employed.[12] The auxiliary plane wave basis sets were truncated at 400 Ry.

Finally, the uncoordinated formates of the Zr_6 Clusters were replaced by OH-H_2O pairs which turns the system into *I*23 (ITA number 197) symmetry. The structure model has been validated on the basis of excellent match of the simulated PXRD pattern of the geometry-optimized structure with the corresponding experimental data (Supplementary Fig. 2e).

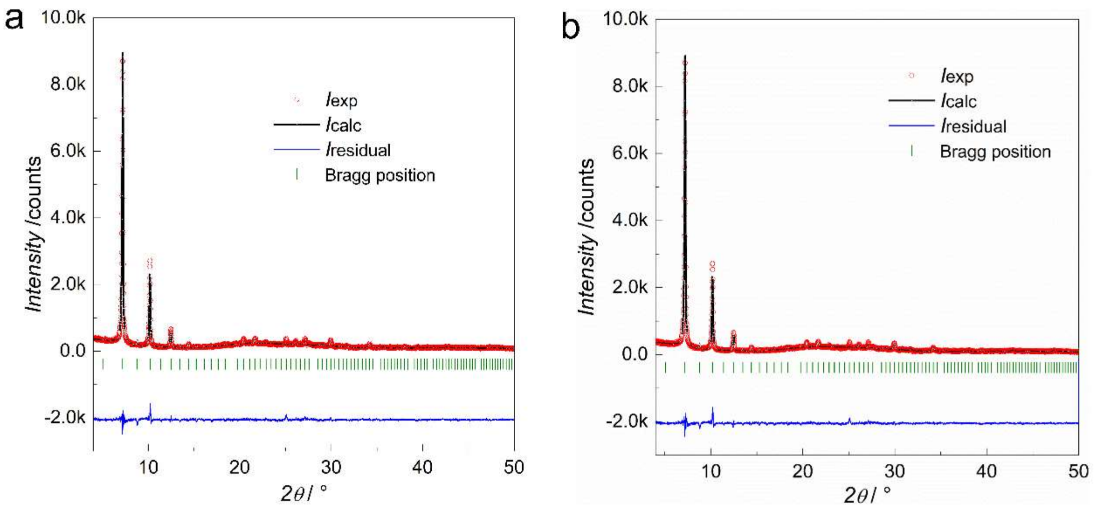

Supplementary Figure 3. Crystal structure refinements using Rietveld method. (a) MIP-201 model with formate molecules coordinated to Zr-oxoclusters: (space group Im-3; RI(Bragg) = 7.85 %; Rwp = 10.8 %; a = 24.584(1) Å); (b) MIP-201 model with hydroxyl and water molecules coordinated to Zr-oxoclusters: (space group Im-3; RI(Bragg) = 7.90 %; Rwp = 10.1 %; a = 24.584(1) Å).

MIP-201 sample was grinded and the fine powder was loaded to 0.7 mm glass capillary. The sample in capillary was measured using Debye-Scherrer geometry with Empyrean 2 diffractometer from Malvern Panalytical, which is equipped with Ge monochromator providing $CuK\alpha_1$ radiation (λ = 1.540598 Å). In order to reduce the noise to intensity ratio, 25 independent data sets were collected and merged in Data Viewer program. The resulting powder diffraction pattern was refined in Fullprof applying Coefficient for monochromatic polarization correction (Cthm = $\cos^2\alpha$ = 0.78964, where α = 27.3° for Ge)[13].

Two structural models have been probed for the refinement (Supplementary Fig. 3). The first model suggests the coordination of 6 formates (HCOO−) and three mdip linkers to $Zr_6O_4(OH)_4^{12+}$ oxoclusters, while the second proposes the coordination of water molecules and hydroxyl groups instead of formates. The checkCIF procedure (ADDSYM) suggested a higher symmetry with *Im-3* space group instead of the initially *I32* one. Such approach significantly reduced the number of the independently refined atomic coordinates and improved the parameters/number of the observed peaks ratio.

The refinement of both models in *Im-3* space group gives a similar fit, however, in case of coordinated water molecules and hydroxyl groups (named as O5A and O5B) the presence of extra water (Ow2) molecules is necessary in order

to make the refinement stable. The obtained Ow2−O5A and Ow2−O5B interatomic distances (1.69(1) and 1.91(1) Å, respectively) suggests, that most likely the formate groups are present in the structure rather than hydroxyl and water: the refinement with O5A−C5 and O5A−C5 soft distance/angle constrains (Supplementary Fig.3 and Table 1) gives the refinement fit with a marginal difference. The refinement of both structures were performed using the soft angle and distance constrains for atomic positions in $Zr_6O_4(OH)_4^{12+}$ oxoclusters and formates, while the atoms of mdip were fixed. The corresponding soft distance and angle constrains are given in Supplementary Table 1. The atomic positions of solvent Ow1 molecules were localized using direct space methods in FOX[14] and refined independently. The atomic displacement parameters were refined isotropically for Zr atoms, while for the lighter C and O atoms they were refined as a single parameter. For the final fit, they all were fixed and hydrogen atoms received 1.25*Biso from C/O atoms.

Supplementary Table 1. The details of the soft distance and angle constrains, applied for the refinement of the structural models of MIP-201

Atom 1	Atom 2	Atom 2	Distance (standard deviation of the distance, sigma), Å	Angle (standard deviation of the angle, sigma), °
Model (2) with hydroxyl and water				
Zr1	O1		2.06(1)	
Zr1	O2		2.06(1)	
Zr1	O5A (formate)		2.25(1)	
Zr1	O5B (formate)		2.25(1)	
C5 (formate)	O5A (formate)		1.28(1)	
C5 (formate)	O5A (formate)		1.28(1)	
C5 (formate)	H5 (formate)		1.14(1)	
O5A (formate)	O11B (formate)		2.45(1)	
Zr1	C5 (formate)		3.20(1)	
Zr1	O1	Zr1		102.0(3)
Zr1	O2	Zr1		102.0(3)
O5A	C5	O5B		120.0(3)
H5	C5	O5A		120.0(3)
H5	C5	O5B		120.0(3)
Model (2) with hydroxyl and water				
Zr1	O1		2.06(1)	
Zr1	O2		2.06(1)	
Zr1	O5A (hydroxyl/water)		2.25(1)	
Zr1	O5B (hydroxyl/water)		2.25(1)	
O5A (hydroxyl/water)	O11B (MDIP)		2.85(1)	
O5B (hydroxyl/water)	O11A (MDIP)		2.85(1)	
O5A (hydroxyl/water)	O11B (hydroxyl/water)		2.45(1)	
Zr1	O1	Zr1		102.0(3)
Zr1	O2	Zr1		102.0(3)

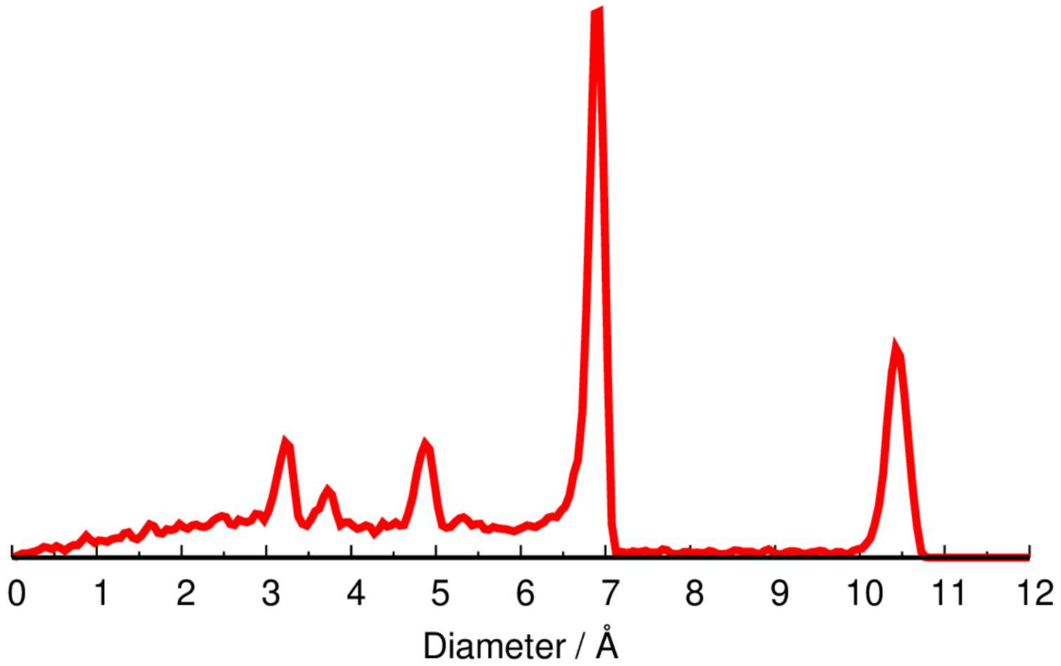

Supplementary Figure 4. Simulated pore size distribution of the MIP-201 DFT optimized geometry.

Pore size distribution calculations. The methodology reported by Gelb and Gubbins[15] was used to calculate the pore size distributions (PSD) of the MIP-201 DFT-optimized structure. In this calculation, the van der Waals parameters of the framework atoms were adopted from Universal Force Field (UFF).[16]

Accessible surface area and Pore volume. The theoretical accessible surface areas (S_{acc}) of the geometric topology of the MIP-201 were calculated using a simple Monte Carlo integration technique where the center of mass of the probe molecule with hard sphere is "rolled" over the framework surface.[15] In this method, a nitrogen sized (3.64 Å) probe molecule is randomly inserted around each framework atoms and the fraction of the probe molecules without overlapping with the other framework atoms is then used to calculate the accessible surface area. The Lennard-Jones size parameters of the framework atoms were also taken from UFF.[16] The free pore volume (V_{pore}) of MIP-201 framework were further calculated using the same geometric method but with a probe molecule of 0 Å.[17]

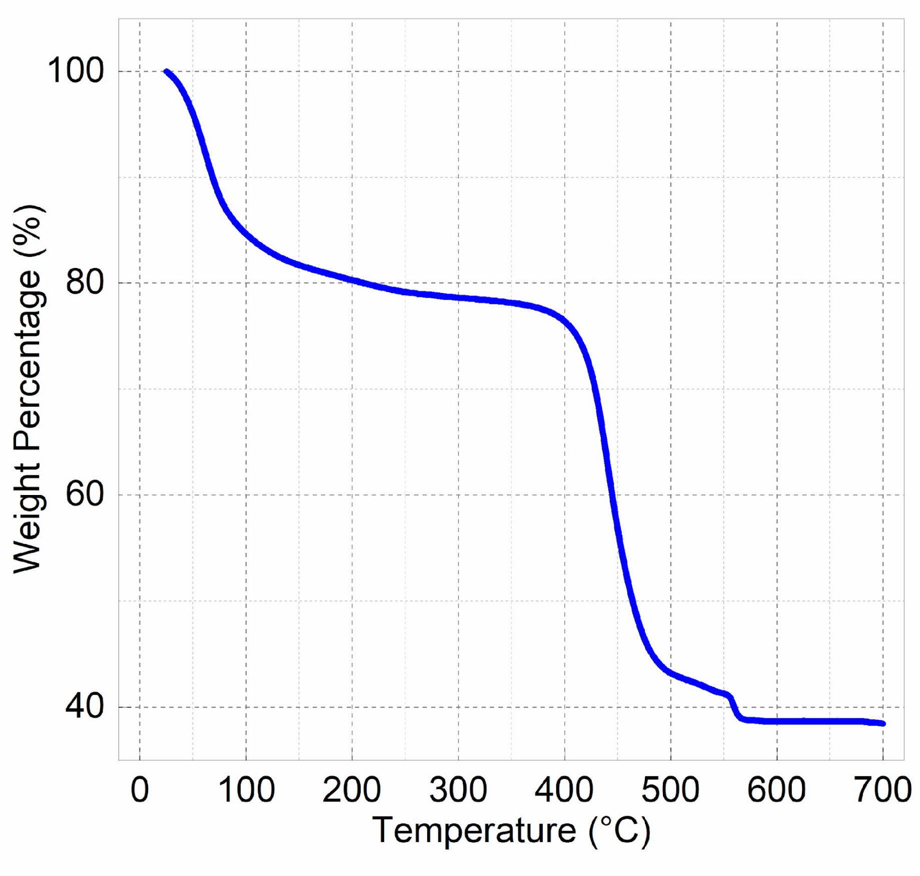

Supplementary Figure 5. TGA curve of MIP-201 product obtained under reflux condition after washing with ethanol and water.

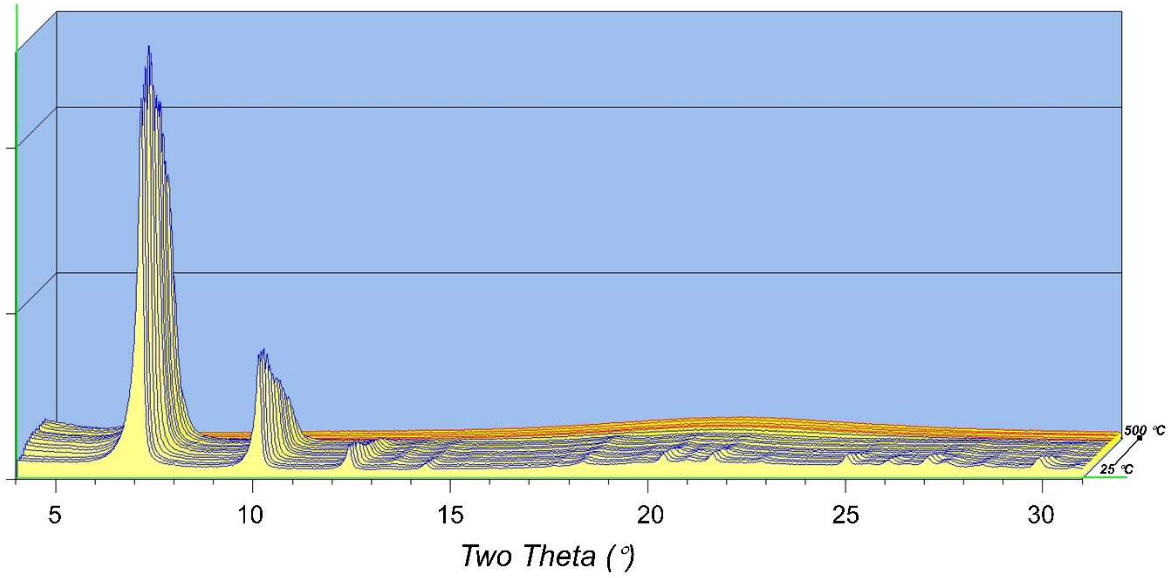

Supplementary Figure 6. Temperature dependent PXRD patterns of MIP-201 product obtained under reflux condition after washing with ethanol and water. Data collection were performed every 25 °C in the temperature range from 25 °C to 500 °C with MIP-201 sample sealed in a quartz capillary in air.

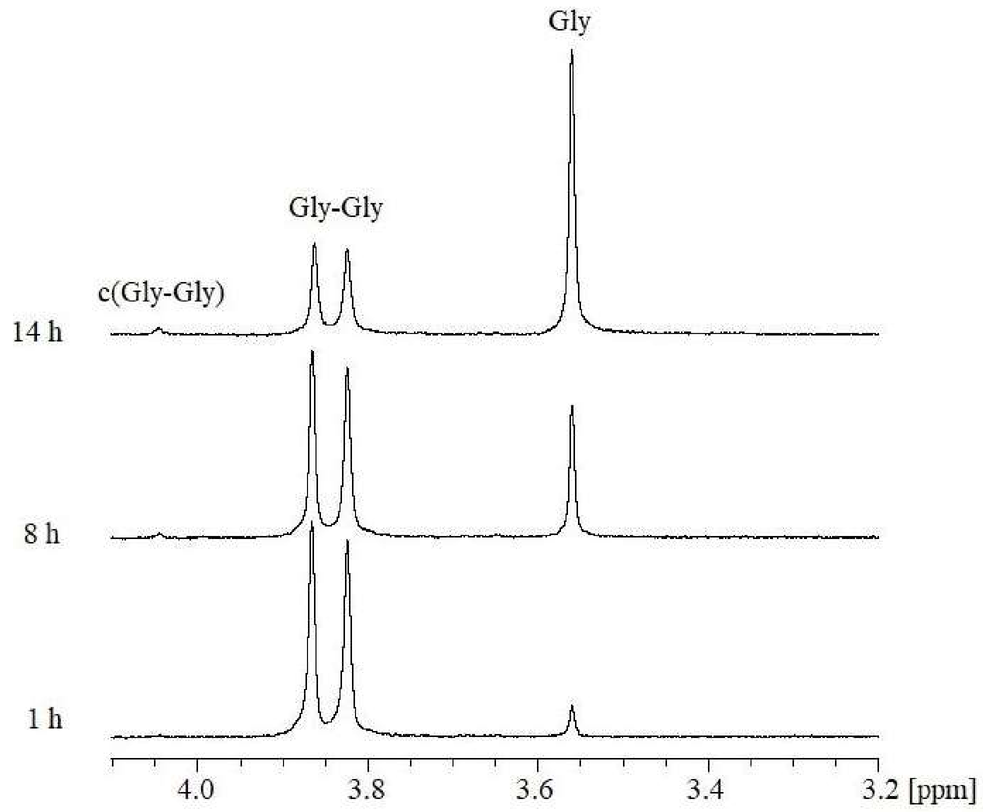

Supplementary Figure 7. ^{1}H NMR spectra recorded at different reaction time during the hydrolysis of 2.0 *µ*mol of Gly-Gly by 2.0 *µ*mol of MIP-201 at pD 7.4 and 60 °C.

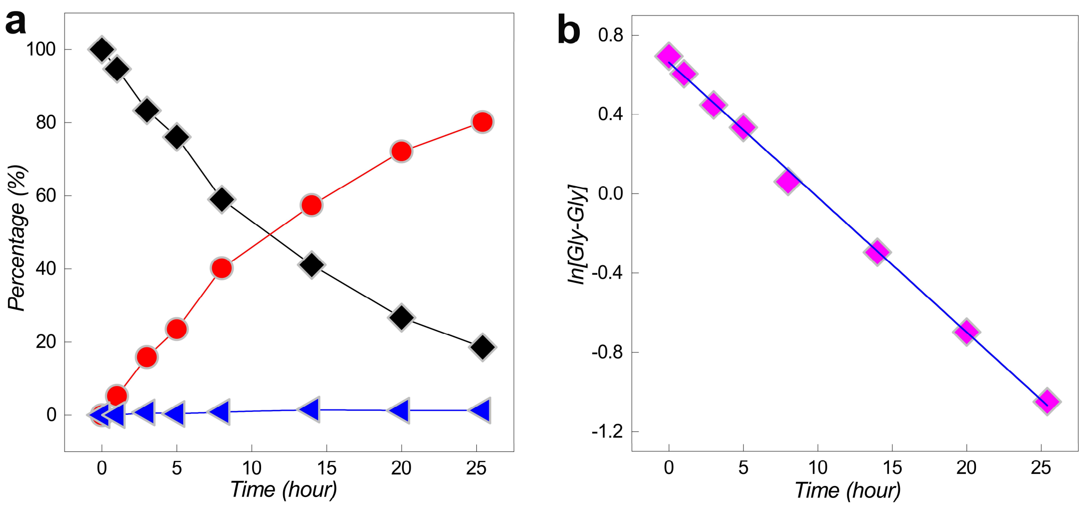

Supplementary Figure 8. Catalytic performance of MIP-201 for hydrolysis of Gly-Gly. a. Percentage of Gly-Gly (◆black square), c(Gly-Gly) (▲blue triangle), and Gly (●red circle) as a function of reaction time for the hydrolysis of 2.0 *μ*mol of Gly-Gly by 2.0 *μ*mol of MIP-201 at pD 7.4 and 60 °C. **b**. ln[Gly-Gly] as a function of time for the reaction of 2.0 *μ*mol of Gly-Gly hydrolysis promoted by 2.0 *μ*mol of MIP-201 at pD 7.4 and 60 °C.

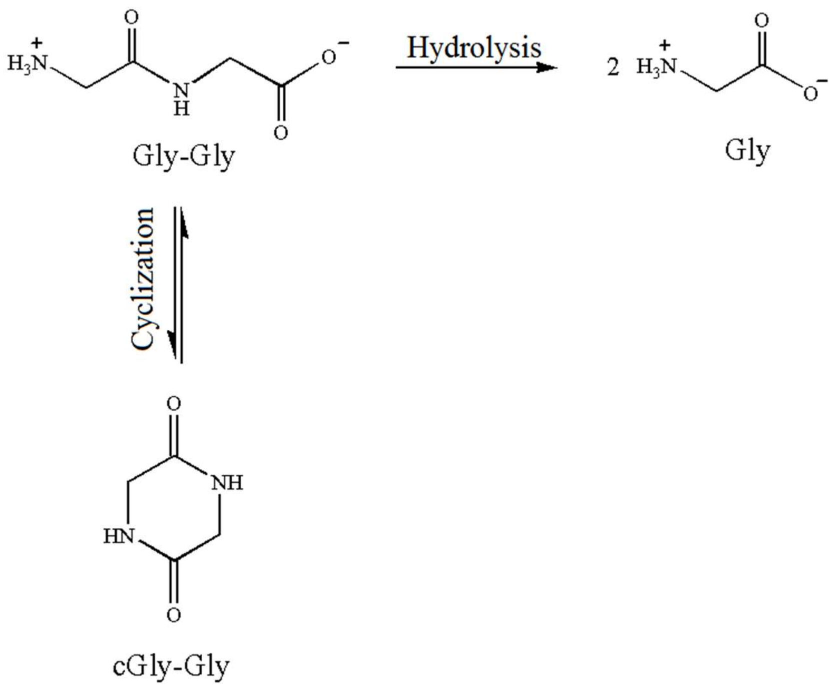

Supplementary Figure 9. Hydrolysis of Gly-Gly and its cyclization to cGly-Gly by MIP-201.

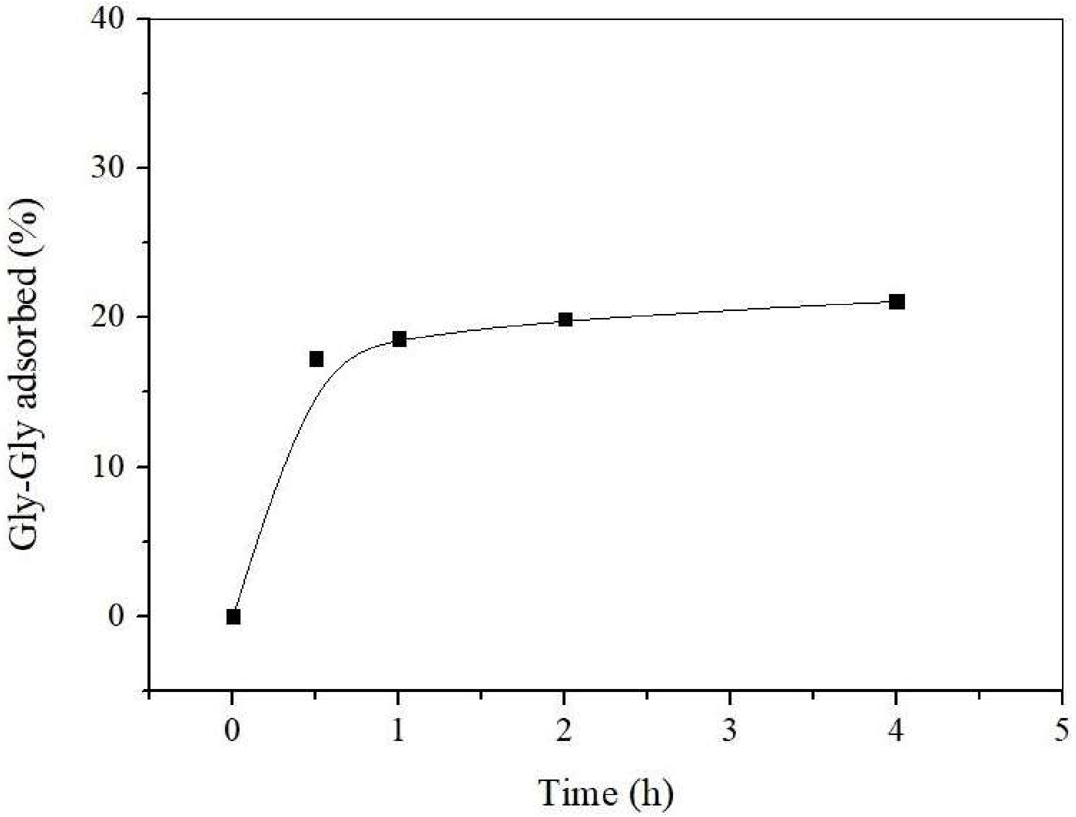

Supplementary Figure 10. The effect of time on the quantity of Gly-Gly adsorbed in MIP-201 at pD 7.4 and room temperature.

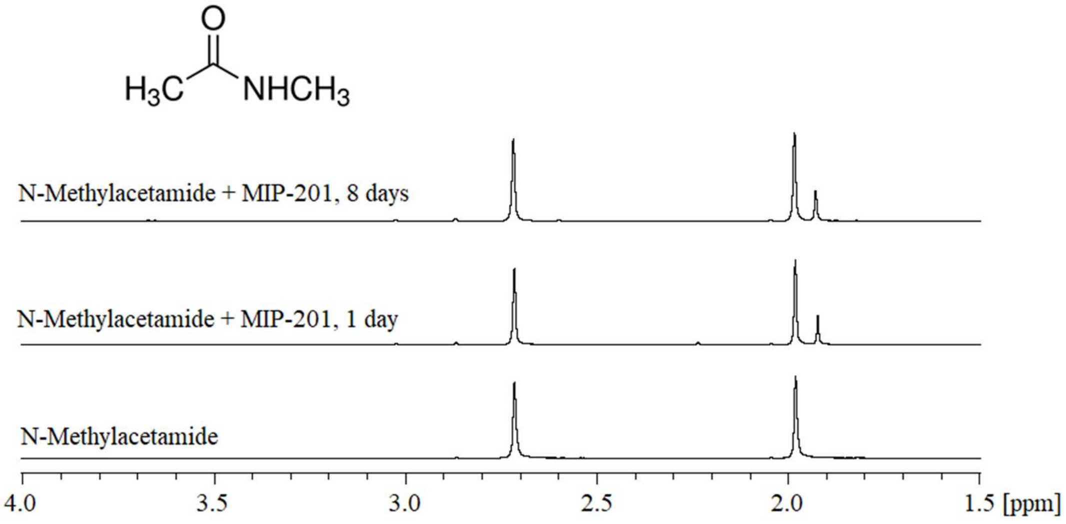

Supplementary Figure 11. Control experiment of hydrolysis of 2.0 *μ*mol N-methylacetamide in the presence of 2.0 *μ*mol MIP-201 at pD 7.4 and 60 °C.

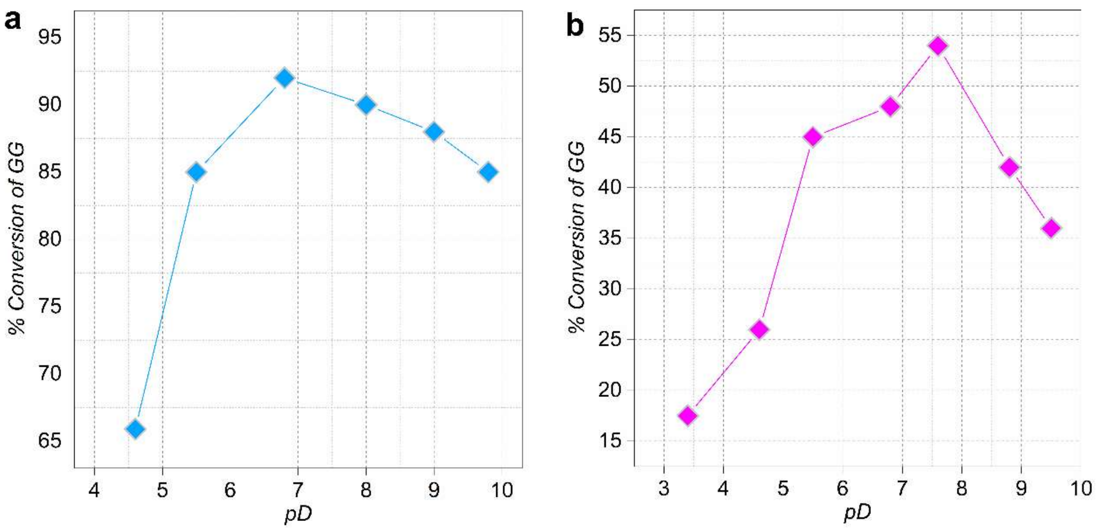

Supplementary Figure 12. (a) Conversion of Gly-Gly after 4 h in the reaction mixtures containing 2.0 *μ*mol of Gly-Gly and 2.0 *μ*mol of MOF-808 at 60 °C and different pD values. (b) Influence of pD on conversion of 2 mM GG with 2 *μ*mol NU-1000, 60 °C, 3 days.

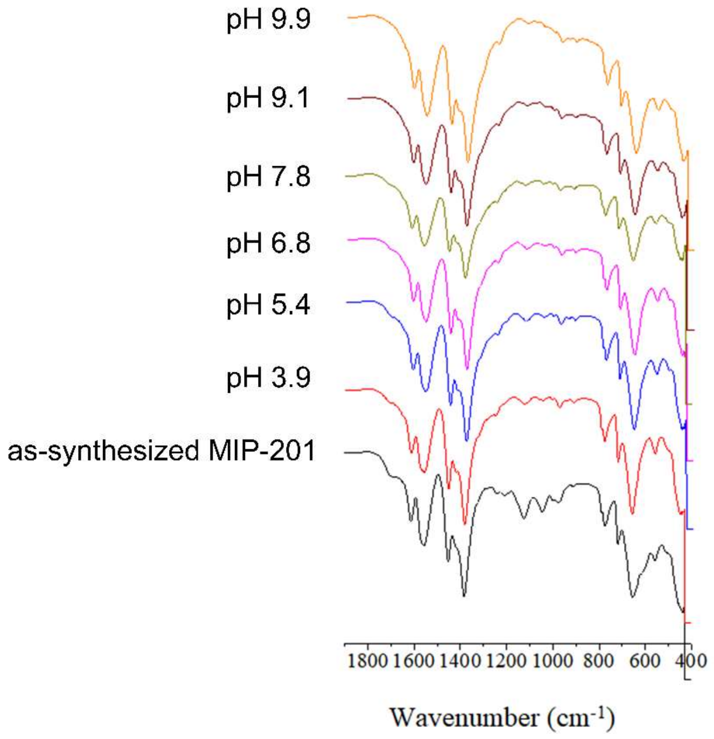

Supplementary Figure 13. FTIR spectra of MIP-201 after 8 h at 60 °C in the reaction mixtures containing Gly-Gly at different pD values.

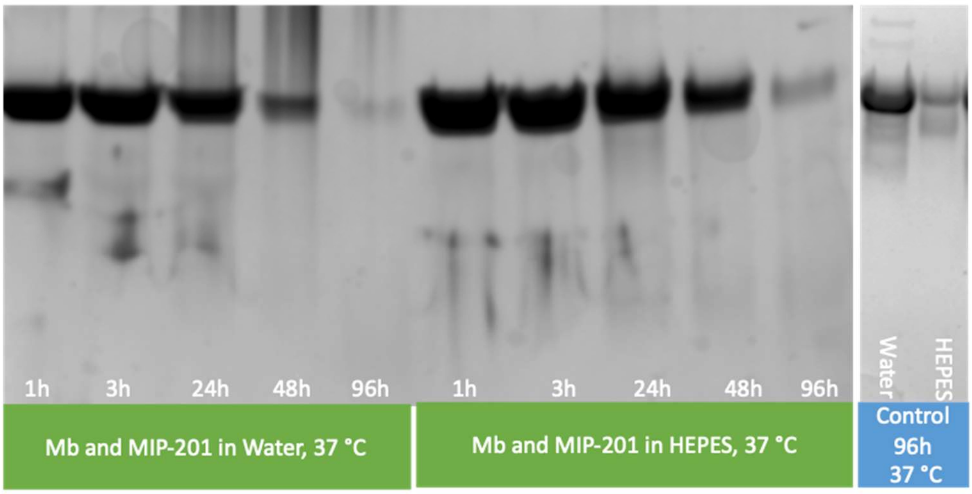

Supplementary Figure 14. SDS PAGE of a sample containing 0.02 mM Mb and 2 μmol MIP-201, incubated at 37 °C in water at pH 7.4 or 0.1M HEPES buffer pH 7.4.

Supplementary Table 2: The intensity of different fragments (in %) observed in Mb hydrolysis upon incubation with MIP-201 at 37 °C in water at pH 7.4 or 0.1M HEPES buffer pH 7.4.

	**Water 37 ℃**					**HEPES 37 ℃**				
**Molecular Weight of Fragment (kDa)**	1h	3h	24h	48h	96h	1h	3h	24h	48h	96h
**16.3**	65.7	63.1	46.2	100.0	100.0	100	100	100.0	100.0	100.0
**12.6**	34.3	18.9	31.6							
**11.2**		18								
**10.6**			22.2							
**Hydrolyzed**	34%	37%	54%	0%	0%	0%	0%	0%	0%	0%

Supplementary Table 3: Analysis of Mb fragments observed in SDS PAGE at different fragments and the proposed cleavage sites.

Fragments formed at 60 °C (kDa)	Fragments formed at 37 °C (kDa)	Hydrolysis Site 1	Hydrolysis Site 2
14.9		Asp20 - Ile21	
13.6		Asp122 - Phe123	
12.5	12.5	Asp109 - Ala110	
11.0	11.2	Asp20 - Ile21	Asp122 - Phe123
10.1	10.5	Asp60 - Leu61	
7.10		Asp60 - Leu61	Asp126 - Ala127

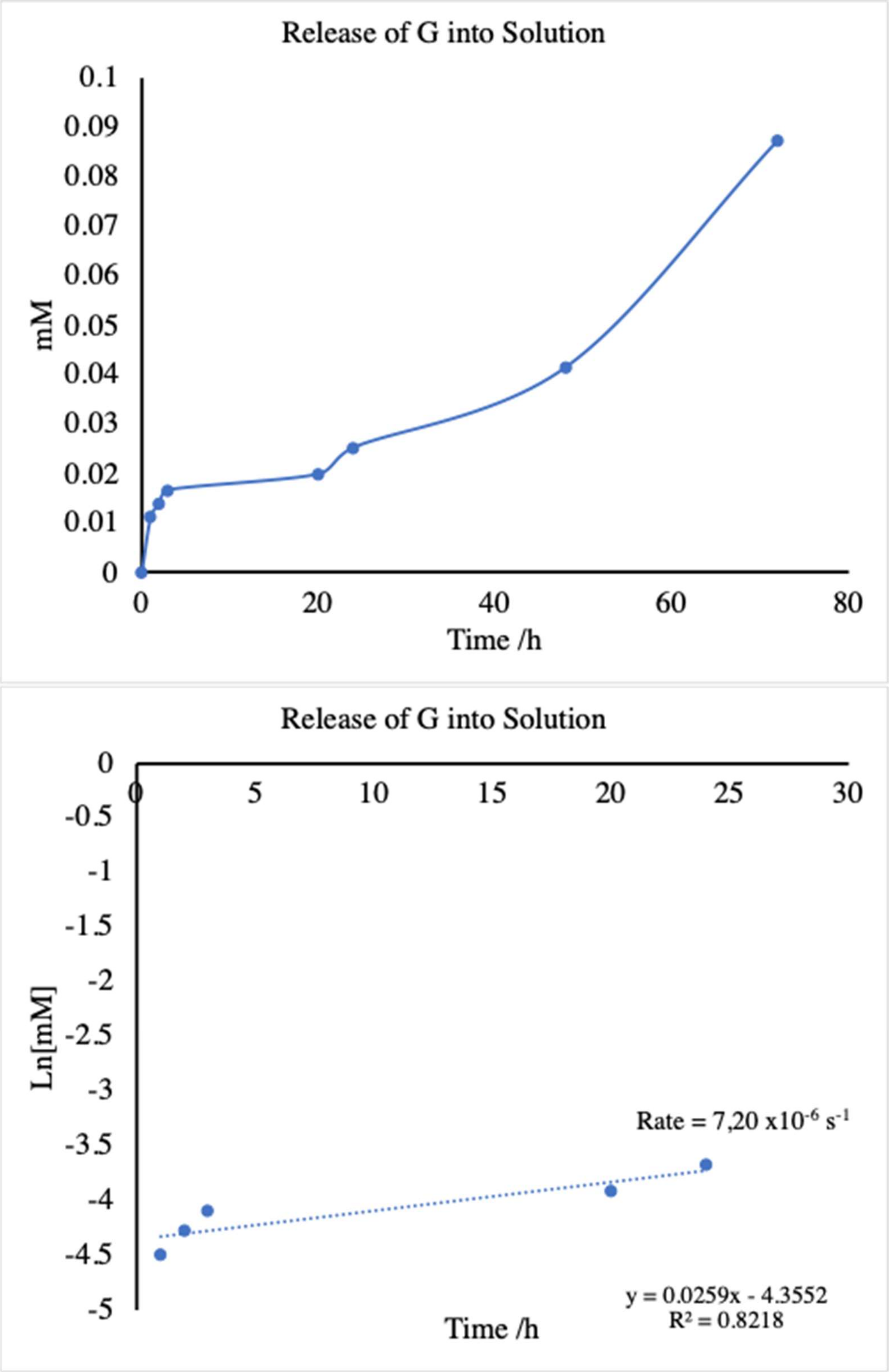

Supplementary Figure 15. Hydrolysis of 2mM Asp-Gly and 2 μmol MIP-201, 60 °C, D_2O.

Rate of G release into solution resulted in k_{obsd}=7.20 x10^{-6} s^{-1}.

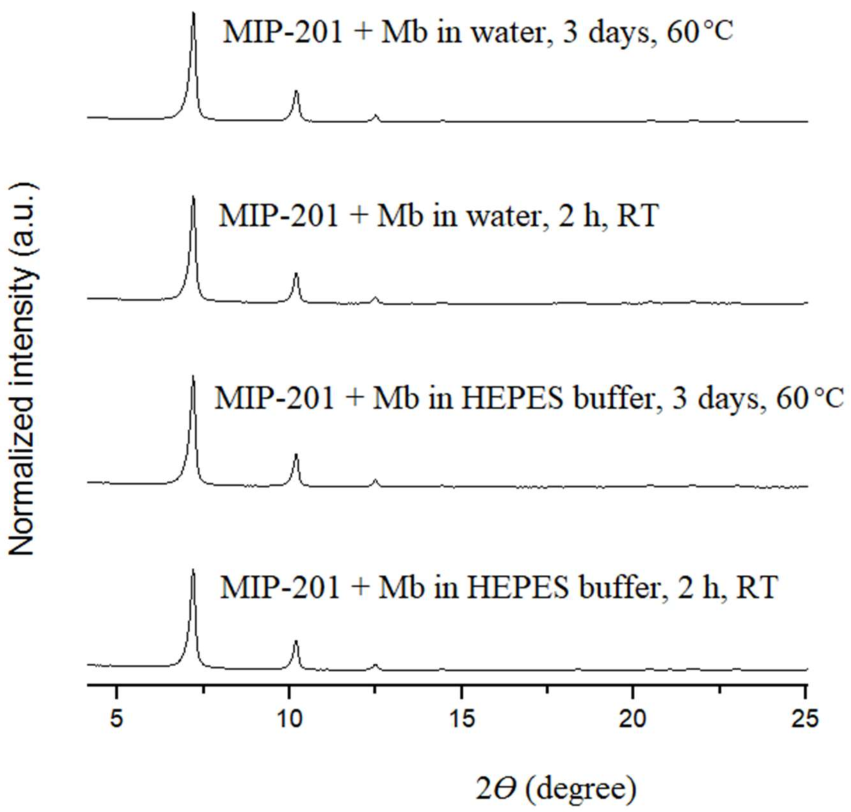

Supplementary Figure 16. PXRD patterns of MIP-201 in the presence of Mb at RT or 60 °C and pH 7.4.

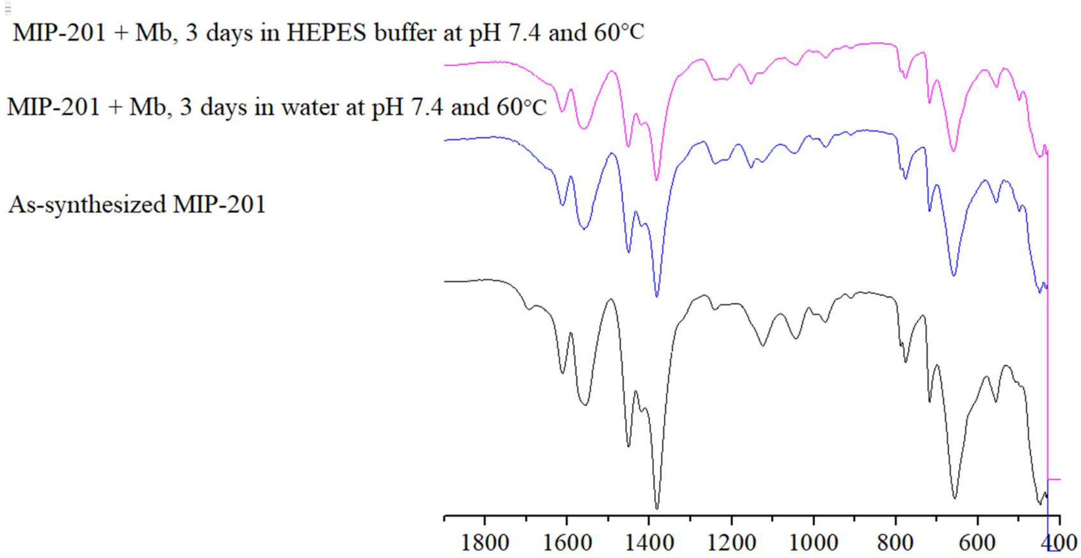

Supplementary Figure 17. FTIR spectra of MIP-201 before and after the hydrolysis of Mb at 60 °C and pH 7.4

Supplementary References

1. Gomez-Gualdron, D.A. *et al.* Computational Design of Metal–Organic Frameworks Based on Stable Zirconium Building Units for Storage and Delivery of Methane. *Chem. Mater.* **26**, 5632-5639 (2014).

2. Wang, S. *et al.* A robust large-pore zirconium carboxylate metal–organic framework for energy-efficient water-sorption-driven refrigeration. *Nat. Energy* **3**, 985-993 (2018).

3. VandeVondele, J. *et al.* Quickstep: Fast and accurate density functional calculations using a mixed Gaussian and plane waves approach. *Comput. Phys. Commun.* **167**, 103-128 (2005).

4. Hutter, J., Iannuzzi, M., Schiffmann, F. & VandeVondele, J. cp2k: atomistic simulations of condensed matter systems. *WIREs Computat. Mol. Sci.* **4**, 15-25 (2014).

5. The CP2K developers group. http://www.cp2k.org.

6. Perdew, J.P., Burke, K. & Ernzerhof, M. Generalized Gradient Approximation Made Simple. *Phys. Rev. Lett.* **77**, 3865-3868 (1996).

7. Grimme, S., Antony, J., Ehrlich, S. & Krieg, H. A consistent and accurate ab initio parametrization of density functional dispersion correction (DFT-D) for the 94 elements H-Pu. *J. Chem. Phys.* **132**, 154104 (2010).

8. Grimme, S. Accurate description of van der Waals complexes by density functional theory including empirical corrections. *J. Comput. Chem.* **25**, 1463-1473 (2004).

9. Goedecker, S., Teter, M. & Hutter, J. Separable dual-space Gaussian pseudopotentials. *Phys. Rev. B* **54**, 1703-1710 (1996).

10. Krack, M. Pseudopotentials for H to Kr optimized for gradient-corrected exchange-correlation functionals. *Theor. Chem. Acc.* **114**, 145-152 (2005).

11. Hartwigsen, C., Goedecker, S. & Hutter, J. Relativistic separable dual-space Gaussian pseudopotentials from H to Rn. *Phys. Rev. B* **58**, 3641-3662 (1998).

12. VandeVondele, J. & Hutter, J. Gaussian basis sets for accurate calculations on molecular systems in gas and condensed phases. *J. Chem. Phys.* **127**, 114105 (2007).

13. Rodriguezcarvajal, J. Recent advances in magnetic-structure determination by neutron powder diffraction. *Physica B* **192**, 55-69 (1993).

14. Favre-Nicolin, V. & Cerny, R. FOX, `free objects for crystallography': a modular approach to ab initio structure determination from powder diffraction. *J. Appl. Crystallogr.* **35**, 734-743 (2002).

15. Gelb, L.D. & Gubbins, K.E. Pore size distributions in porous glasses: A computer simulation study. *Langmuir* **15**, 305-308 (1999).

16. Rappe, A.K., Casewit, C.J., Colwell, K.S., Goddard, W.A. & Skiff, W.M. UFF, a full periodic

table force field for molecular mechanics and molecular dynamics simulations. *J. Am. Chem. Soc.* **114**, 10024-10035 (1992).

17. Duren, T., Millange, F., Ferey, G., Walton, K.S. & Snurr, R.Q. Calculating geometric surface areas as a characterization tool for metal-organic frameworks. *J. Phys. Chem. C* **111**, 15350-15356 (2007).
